# Role of Additives: Modified Hemihydrate Phosphogypsum Morphology and Enhanced Filtration Performance of Wet-Process Phosphoric Acid

**DOI:** 10.1021/acsomega.3c08259

**Published:** 2023-12-01

**Authors:** Xuejian Huo, Lanfeng Guo, Renlong Liu, Changyuan Tao, Benjun Xi

**Affiliations:** † College of Chemistry and Chemical Engineering, Chongqing University, Chongqing 400044, China; ‡ State Key Laboratory of Coal Mine Disaster Dynamics and Control, Chongqing University, Chongqing 400044, China; § Hubei Three Gorges Laboratory, Yichang 443007, China

## Abstract

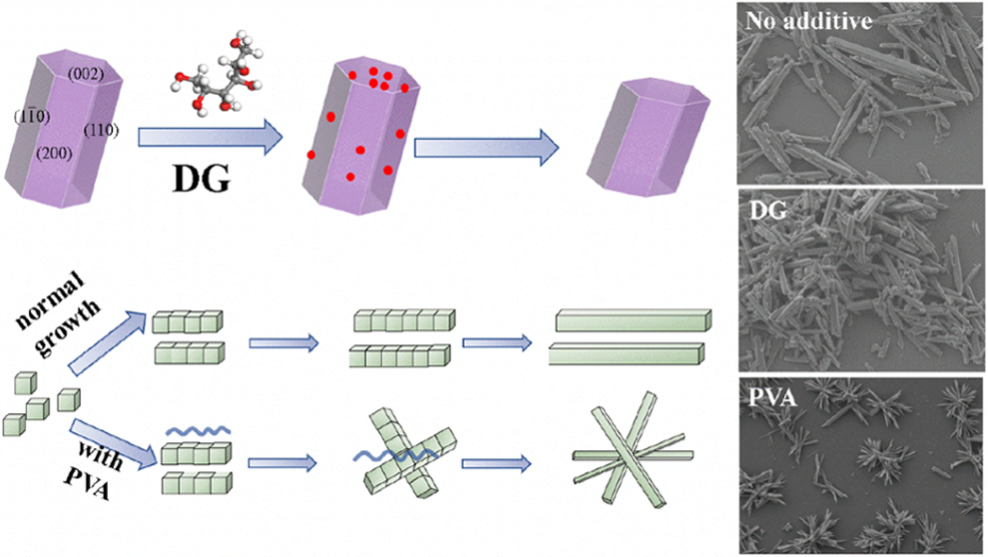

The morphology of hemihydrate phosphogypsum crystals is of vital importance in the hemihydrate–dihydrate (HH–DH) wet-process phosphoric acid production for high filtration strength. The morphology of hemihydrate phosphogypsum is commonly needlelike due to the strong acidic crystallization environment, which is unfavorable to the following filtration process. In this study, the crystal habit of hemihydrate phosphogypsum with a large aspect ratio was skillfully modified by additives to achieve a higher filtration strength. d-Glucitol (DG) reduces the theoretical aspect ratio of hemihydrate phosphogypsum crystals from 2.076 to 1.583 by interacting with the (002) face of CaSO_4_·0.5H_2_O preferentially, and poly(vinyl alcohol) (PVA) facilitates the aggregation of small grains to gather into a clusterlike structure. The modified morphologies of hemihydrate phosphogypsum have a lower bulk density and a larger porosity of the formed filter cake, which increases the filtration strength up to 45.9% when DG is added. Our work provides an in-depth explanation of the evolution mechanism of hemihydrate phosphogypsum morphology with the additives and its influence on the filtration performance. The improved filtration strength would reduce the water content of hemihydrate phosphogypsum and relieve the storage pressure of the phosphogypsum slag dump, which is meaningful to the clean production and process emission reduction of the phosphorus chemical industry.

## 1 Introduction

Sulfuric acid-based wet-process phosphoric acid (WPA) is a significant method for the production of phosphoric acid.^1−3^ Compared to the traditional dihydrate (DH) method, the hemihydrate–dihydrate (HH–DH) method^4,5^ serves as a new way to produce phosphoric acid along with an intermediate product of hemihydrate phosphogypsum (CaSO_4_·0.5H_2_O as the main composition) rather than directly generating dihydrate phosphogypsum and giving an opportunity to produce a higher content of P_2_O_5_ (≥40 wt %) without an energy-intensive concentration process.^6^ The reaction process of the HH–DH method contains the dissolution of phosphate ore and the subsequent hydration of hemihydrate phosphogypsum to dihydrate phosphogypsum, as represented in the following reactions:
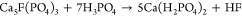
1
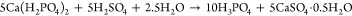
2
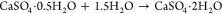
3

The obtained hemihydrate phosphogypsum in the leaching process needs to be filtered before being transferred to a hydration tank to transform it into dihydrate phosphogypsum. However, the filtration strength of slurries is not high enough owing to the small size and high aspect ratio of hemihydrate phosphogypsum, which is unfavorable to the formation of an effective filtering path and even causes the filter cake to crack and the filtration to be short-circuited. On the other hand, pH plays a decisive role in the morphology of calcium sulfate hydrates,^7−9^ and phosphogypsum with a high aspect ratio is commonly obtained owing to the strong acidity condition of WPA, which significantly decreases the filtration strength.^10^ Moreover, the filtration pore may even be blocked during the filtration process owing to the needle-shaped phosphogypsum, which increases the load of the filter and causes the plugging of the filter.^11^ So it is of vital importance to modify the crystal habit of phosphogypsum crystals during WPA to achieve a higher filtration rate.^11−16^ In such a strong acidic environment, additives become an attractive way to modify the morphology of hemihydrate phosphogypsum. Commonly, additives have a negative effect on crystallization, which can be well described by the selective adsorption on the nucleation and growth site,^17−19^ and inhibit the crystal growth rate, leading to a smaller size or a modified habit.

Some studies have concentrated on the influence of additives on phosphogypsum crystallization and filtration strength in the past. Titiz-Sargut et al.^11^ found that the average particle size of gypsum was reached to maximum, and the minimum cake resistance and maximum filtration rate were obtained in the presence of 2500 ppm citric acid concentration during the crystallization of calcium sulfate dihydrate. Rashad et al.^20^ studied the effect of Al^3+^ and Mg^2+^ on the crystallization of dihydrate phosphogypsum and interestingly found that the crystal’s mean and median diameters increased in the presence of Al^3+^ and decreased in the presence of Mg^2+^, respectively. Mahmoud et al.^21^ investigated the effect of CTAB and SDS on the crystallization of dihydrate phosphogypsum and found that the percentage of fine crystals decreased in the presence of CTAB and increased in the presence of SDS. Kruger et al.^12^ showed that impurity ions except for F^–^ aided phosphogypsum precipitation and the filtration rates decrease at higher impurity levels. Abdel-Aal et al.^16^ studied the effect of CMR-100 on the phosphogypsum filtration rate and a 48% increase in the filtration rate was achieved. Regretfully, none of the above studies gave a deep understanding of the crystal size and habit change mechanism of phosphogypsum during the leaching process and its effects on filtration strength.

There have also been some studies on the role of metal ions and surfactants in the crystal habit of CaSO_4_·0.5H_2_O. Hou et al.^22^ found that the presence of Mg^2+^ led to the increase in the aspect ratios of α-CaSO_4_·0.5H_2_O whiskers, which can be ascribed to the doped Mg^2+^ adsorption on the side surfaces of CaSO_4_·0.5H_2_O, and inhibited the growth of these facets and promoted the 1-D growth along the *c* axis. Fan et al.^23^ found that the shape of CaSO_4_·0.5H_2_O changed from whiskers to short rodlike after adding Al^3+^. Li et al.^24^ studied the influence of aluminum on CaSO_4_·0.5H_2_O crystallization and found that Al^3+^ retards the nucleation rates by increasing the interfacial tension values. Mao et al.^25^ found that the addition of CTAB made smaller calcium sulfate whiskers with high aspect ratios. Yang et al.^26^ investigated the effects of metal ions on the crystallization of CaSO_4_·0.5H_2_O under simulated conditions of WPA and showed that the shape of α-HH crystals changes from needlelike to wedge-shaped or short columns in the presence of Mg^2+^, Al^3+^, and Fe^3+^. However, the growth environment of phosphogypsum crystals is more complex during WPA, and the effect of additives on hemihydrate phosphogypsum crystallization and the mechanism behind it are worth studying.

The present work aims to enhance the filtration strength of hemihydrate phosphogypsum obtained in the first stage of HH–DH WPA by controlling the crystal morphology. The crystal habit of hemihydrate phosphogypsum was modified, and the filtration strength was enhanced by adding PVA and DG to the leaching system. And the evolution mechanism of the crystal habit of the phosphogypsum crystal under different conditions was explored.

## 2 Experimental Section

### 2.1 Materials

The phosphate ore raw material was provided by Hubei Xingfa Chemicals Group Co., Ltd., China. It was heated to remove moisture at 60 °C in a drying oven and then ground to a fineness of 75 μm before the experiments. All of the chemical reagents used in this study were of analytical grade, and the experimental water was ultrapure water.

### 2.2 Simulated Hemihydrate Leaching Process of WPA

75 g of grounded phosphate ore was dissolved in a 300 mL solution containing 38 wt % P_2_O_5_ and the mixture was heated to 95 °C using a thermostatically controlled water bath to simulate the industrial wet-process phosphoric acid production process. Then, 30 mL of concentrated sulfuric acid (18.4 mol/L) was added to the heated solutions with a stirring rate of 100 rpm with a rigid-flexible combined impeller^27^ at the temperature of 95 °C. After 120 min, the slurries were filtered and washed several times and dried for further investigations.

Sodium dodecyl benzenesulfonate (SDBS), d-glucitol (DG), and poly(vinyl alcohol) (PVA) were chosen to study the effect of additives on hemihydrate phosphogypsum crystallization for the representation of ionic surfactants, nonionic surfactants, and polymers, respectively. For each additive, the dosages of 0.01, 0.03, and 0.05 wt % were mixed in the leaching solution before adding sulfuric acid, respectively. And the other experimental steps were the same as those of the blank experiment.

### 2.3 Characterization

The phase composition of leaching phosphogypsum products was analyzed by using an X-ray diffractometer (XRD-6000, Shimadzu Corporation, Japan) using Cu Kα radiation as the X-ray source, and the element composition was characterized by using an X-ray fluorescence (XRF) spectrometer (XRF-1800, Shimadzu Corporation, Japan). The SEM images and EDS patterns were determined by using a scanning electron microscope (SEM) (S-570, Hitachi, Ltd., Japan). Fourier-transform infrared (FTIR) spectra were obtained by using an FTIR spectrometer (IRPrestige-21, Shimadzu Corporation, Japan) with the KBr pellet method. The particle size of phosphogypsum was determined by using a QICPIC/L particle size and shape meter. The bulk density of phosphogypsum was determined by measuring the mass of the leaching residue in a 10 mL cylinder after sufficient shaking. The porosity ε of the filter cake was determined by the methods of the published work.^28^
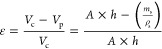
4where *V*_c_ is the volume of the filter cake, *V*_p_ is the volume of phosphogypsum, *A* is the area of the filter cake, *h* is the height of the filter cake, *m*_s_ is the dry mass of phosphogypsum, and ρ_s_ is the solid density of phosphogypsum, which was measured by a gas pycnometer.

Filtration strength measurements were performed under a constant pressure of 0.1 MPa with an SHZ-D circulating water vacuum pump to detect the filtration properties of hemihydrate phosphogypsum. The filtration strength is defined as the mass of phosphogypsum retained per unit area of filter paper per unit of time. The mass of the leaching residue retained on the filter paper was recorded, and three experiments were taken for each sample to obtain the average value. The filtration time is determined by the time elapsed before no obvious liquid can be observed on the filter cake.

### 2.4 MD Simulation

The CaSO_4_·0.5H_2_O crystal structure is obtained from a previous study^29^ with a space group of I121, and the unit cell parameters are *a* = 12.032 Å, *b* = 6.9269 Å, *c* = 12.617 Å, α = γ = 90°, and β = 90.27°.

**Figure 1:**
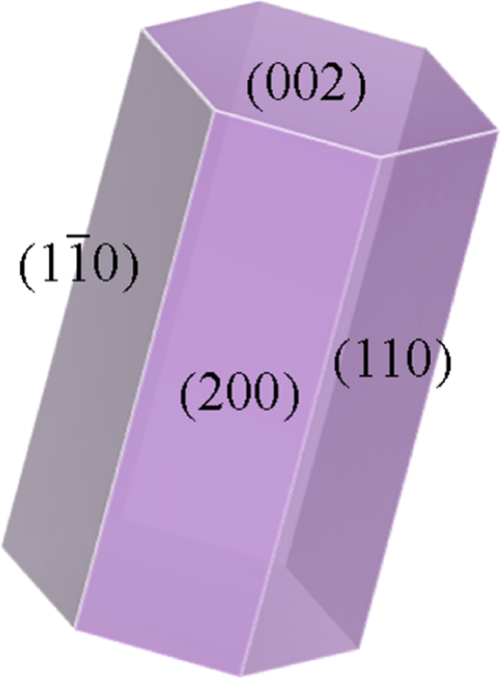
Crystal habit of CaSO_4_·0.5H_2_O based on the attachment energy model.

The theoretical crystal habit of α-CaSO_4_·0.5H_2_O is predicted based on the attachment energy method,^30−32^ and there are four dominant habit faces of the α-CaSO_4_·0.5H_2_O crystal (Figure 1): (200), (110), (1–10), and (002) faces. Both the (200) and (002) crystal surfaces are composed of water molecules, Ca^2+^, and SO_4_^2–^, while the (110) surface is composed of Ca^2+^ and SO_4_^2–^, and the (1–10) surface is composed only of SO_4_^2–^ (Figure S1).

The crystal surface models were constructed with Materials Studio 2019 software with a vacuum slab of 50 Å. The additive molecule was randomly distributed on the cleaved surface to investigate the interaction mechanism of additives on different faces of CaSO_4_·0.5H_2_O. Each system was geometrically optimized through 5000 iterations with a convergence criterion of 1 × 10^–4^ Hartree before MD simulations. MD simulations were performed with the Forcite module of Materials Studio 2019 using the COMPASS force field parametrized with nonzero force field charges. The simulated temperatures were controlled with a Berendsen thermostat set to 373 K to simulate the crystallization temperature of hemihydrate phosphogypsum. The electrostatic interactions and van der Waals forces were calculated by the group-based summation method and the Ewald summation method, respectively. The time step was set to 1 fs. The MD equilibration involved adjusting the density of the simulation system through a 500 ps simulation run with an NVT (constant number of particles, constant volume, and constant temperature) ensemble, followed by a 1000 ps dynamic simulation. During the simulation runs, only the additives were movable, and the crystal surfaces were fixed.

The equilibrated structures were then minimized with a geometry optimization procedure, and the interaction energy between the additives and the crystal surfaces *E*_i_ was obtained by the following expression:
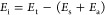
5where *E*_t_ is the total energy of the system (includes all atoms of the crystal surface and the additive molecule), *E*_s_ is the energy of the crystal surface, and *E*_a_ is the energy of the additive molecule.

The modified attachment energy is calculated by the surface docking approach, a simulation procedure developed to predict the influence of additives on the crystal morphology.^33^
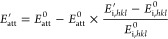
6where *E*_att_^0^ is the attachment energy of the crystal face without additives. And the corresponding crystal habits with the presence of additives are calculated by using the attachment energy model.^30−32^

## 3 Results and Discussion

### 3.1 Leaching Process and the Filtration Strength of Leaching Slurries with Different Additives

**Figure 2:**
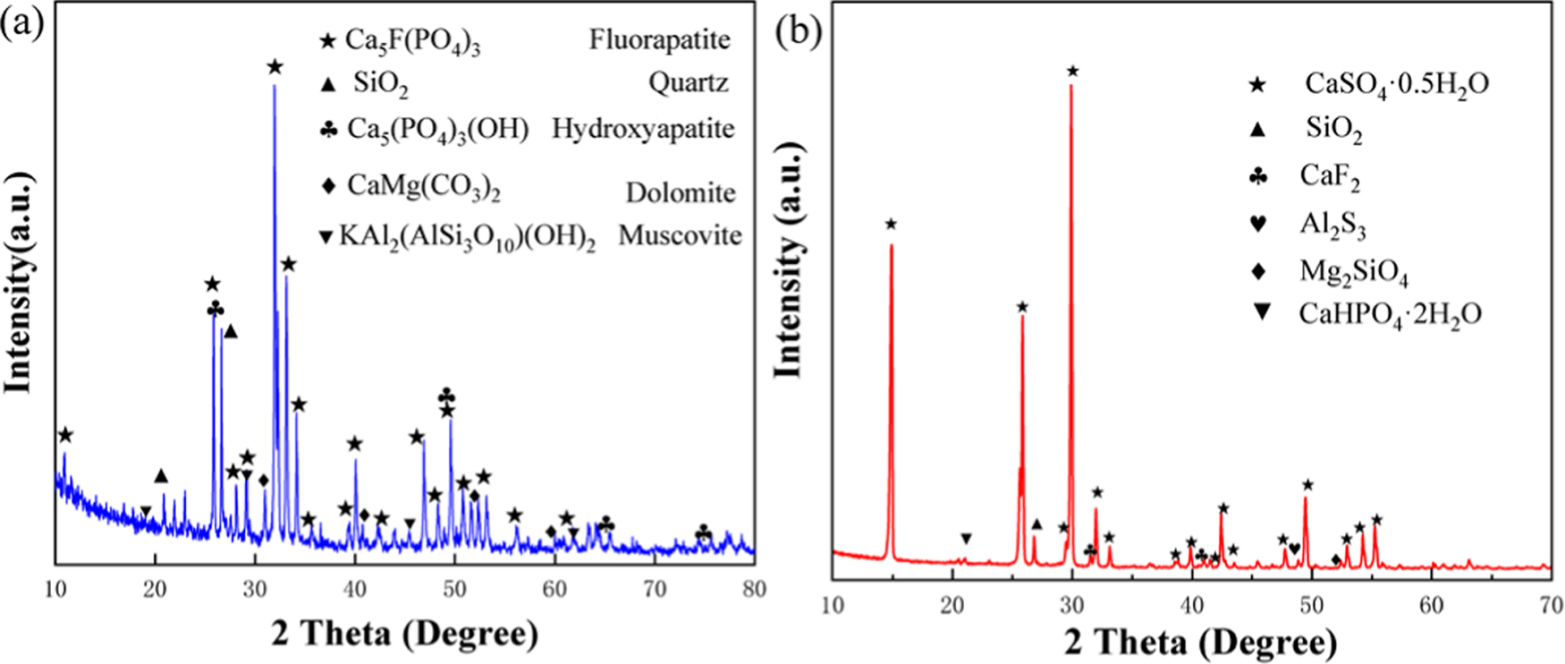
XRD patterns of (a) phosphate ore and (b) leaching hemihydrate phosphogypsum.

The chemical composition of phosphate ore was determined by XRF, and the main element composition is calcium, phosphorus, silicon, aluminum, and so on, as shown in Table 1. The phase composition of phosphate ore was further measured by XRD, and the XRD pattern shown in Figure 2a indicates that the main composition of phosphate ore is Ca_5_(PO_4_)_3_F (PDF No. 15-0876), SiO_2_ (PDF No. 82-1562), Ca_5_(PO_4_)_3_(OH) (PDF No. 09-0432), KAl_2_(AlSi_3_O_10_)(OH)_2_ (PDF No. 07-0025), and CaMg(CO_3_)_2_ (PDF No. 73-2444). After leaching by concentrated H_2_SO_4_, the dominant component of the leaching residue (Figure 2b) turns to CaSO_4_·0.5H_2_O (PDF No. 41-0224), which confirms that the leaching residue is hemihydrate phosphogypsum rather than dihydrate phosphogypsum. There are also some other phases in the leaching residue, CaHPO_4_·2H_2_O, SiO_2_, CaF_2_, Al_2_S_3_, Mg_2_SiO_4_, etc., which are the result of trace impurity elements in phosphate ore.

**Table 1:** XRF Composition Result of Phosphate Ore

element	Ca	P	Si	Al	F	Mg	K	S	Fe	Na
content (%)	33.27	13.53	5.30	1.70	2.37	0.82	1.02	1.10	0.68	0.25

The rapid decomposition of phosphate ore is accompanied by the swift crystallization of phosphogypsum. Hemihydrate phosphogypsum emerges as soon as WPA begins, which is confirmed by the XRD patterns shown in Figure S2, wherein CaSO_4_·0.5H_2_O becomes the domain phase within 2 min of WPA. And the optical images shown in Figure S2 also show that hemihydrate phosphogypsum emerges within only 2 min. The fast crystallization is due to the ultrahigh supersaturation degree of the reaction crystallization system.^34^

**Figure 3:**
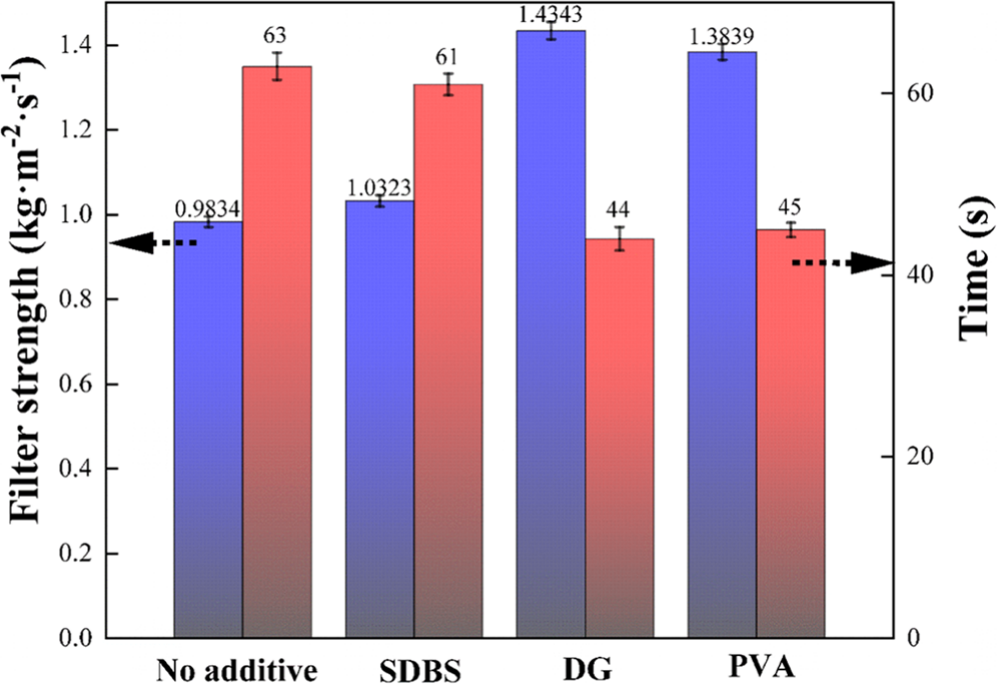
Filtration performance of hemihydrate phosphogypsum with different additives at a dosage of 0.03 wt %.

After the leaching process, a filtration experiment of hemihydrate phosphogypsum at the laboratory level was carried out. Figure 3 shows the filtration strength and filtration time of hemihydrate phosphogypsum with the presence of different additives at a dosage of 0.03 wt %. SDBS provides finite filtration strength improvement, and the addition of DG and PVA significantly reduces the filtration time and increases the filtration strength compared with the phosphogypsum without any additives. DG is regarded as an attractive additive with a filtration strength of 1.4343 kg/m^2^·s and a filtration time of 44 s, which greatly improves the filtration strength compared to the condition of no additives.

### 3.2 Influence of Additives on the Crystallization of Hemihydrate Phosphogypsum

**Figure 4:**
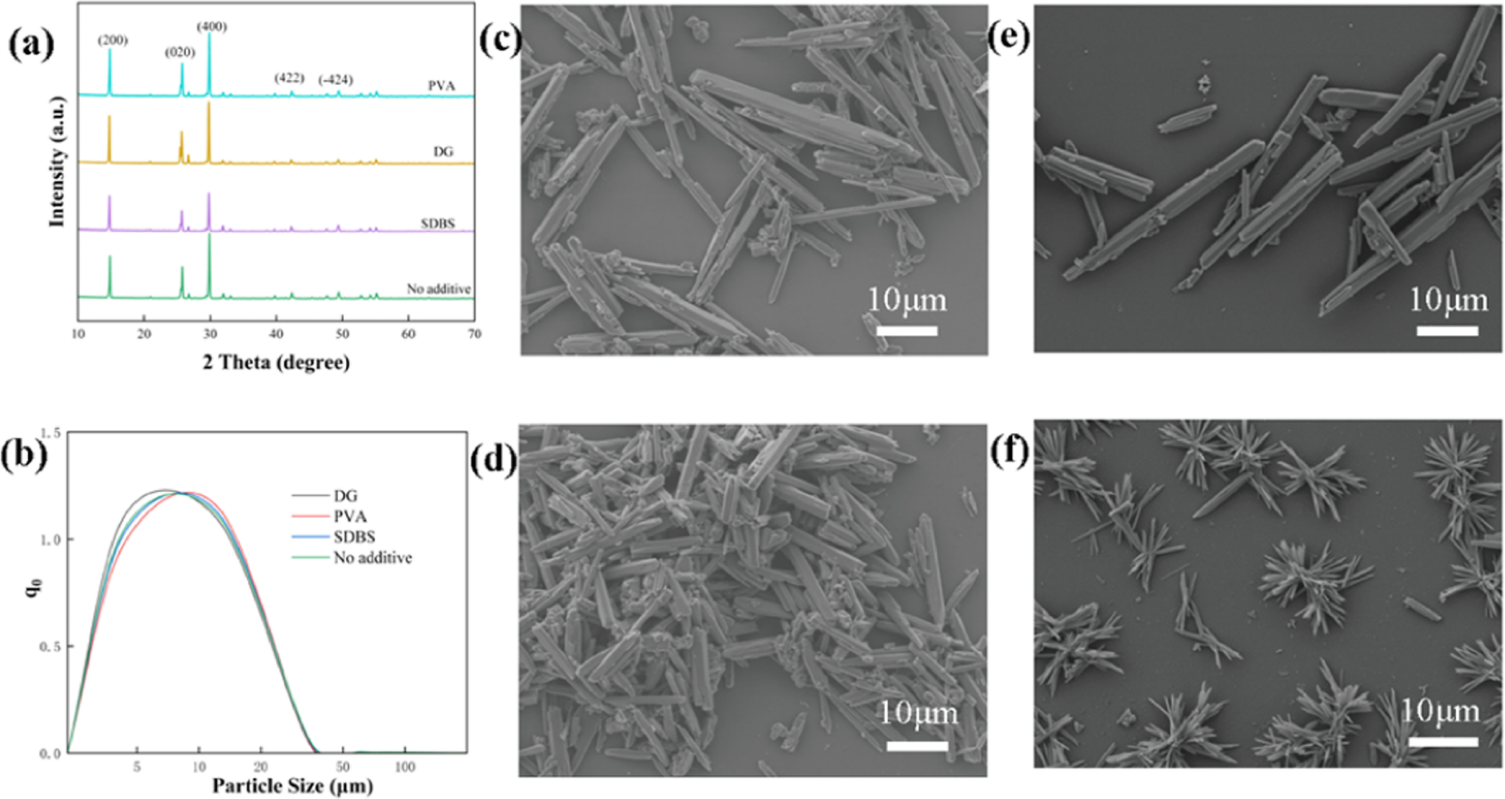
(a) XRD patterns and (b) particle size distribution of the leaching residue obtained with different additives. SEM images of hemihydrate phosphogypsum (c) without additives and with (d) DG, (e) SDBS, and (f) PVA, respectively.

As shown in Figure 4a, the addition of three additives has little impact on the phase of the leaching residue, and all of the patterns show that the main phase of the leaching residue is CaSO_4_·0.5H_2_O, and no additional peaks are observed. As shown in Figure 4c–f, pure hemihydrate phosphogypsum without any additives has a fine needlelike morphology, as observed by SEM photographs and as also confirmed by the calculated crystal habit result with an aspect ratio of 2.076 (Figure 1). The higher aspect ratio of hemihydrate phosphogypsum than the theoretical morphology of CaSO_4_·0.5H_2_O in vacuum can be ascribed to the interaction between the crystal planes of CaSO_4_·0.5H_2_O with the liquid water molecule.^35^ Different morphological changes of hemihydrate phosphogypsum were observed with the presence of different additives. The addition of SDBS has a limited impact on the size and morphology of phosphogypsum (Figure 4e), and phosphogypsum is still needlelike. However, the aspect ratio of hemihydrate phosphogypsum decreases and the particle becomes shorter and coarser when DG is added (Figure 4d). As PVA is added, individual phosphogypsum grains become thicker but aggregate into clusterlike structures (Figure 4f). The agglomeration of the hemihydrate phosphogypsum whiskers gives a smaller aspect ratio and a modified morphology.

The particle sizes of hemihydrate phosphogypsum with different additives are shown in Figure 4b. The size of the obtained hemihydrate phosphogypsum is in the range of 3–24 μm. It is shown that SDBS nearly has no impact on the particle size distribution of hemihydrate phosphogypsum. PVA slightly enlarges the size of hemihydrate phosphogypsum, while DG slightly decreases it, and the effect of additives on the particle size of phosphogypsum can be ignored. It is indicated that the critical role of additives on phosphogypsum is not the crystal size but the crystal habit.

### 3.3 Morphology Evolution Mechanism in the Presence of Additives

To investigate the effect of different additives on the crystal habit of hemihydrate phosphogypsum, molecular dynamics simulation was taken to evaluate the interaction between additives and the four host crystal faces of CaSO_4_·0.5H_2_O, and the results are shown in Table 2. DG and SDBS have strong interactions with the habit face of the CaSO_4_·0.5H_2_O crystal, while PVA nearly has no interaction with any habit face of CaSO_4_·0.5H_2_O and even a positive interaction energy with the (002) face, which indicates that PVA has no potential effect on the crystal habit of CaSO_4_·0.5H_2_O.

**Table 2:** Interaction Energies (kcal/mol) between the Additive and Crystal Face of CaSO_4_·0.5H_2_O

additive	SDBS	DG	PVA
(002)	–705.74	–968.63	57.08
(200)	–746.19	–859.94	–8.38
(110)	–750.18	–747.98	–4.65
(1–10)	–815.36	–860.83	–2.13

**Figure 5:**
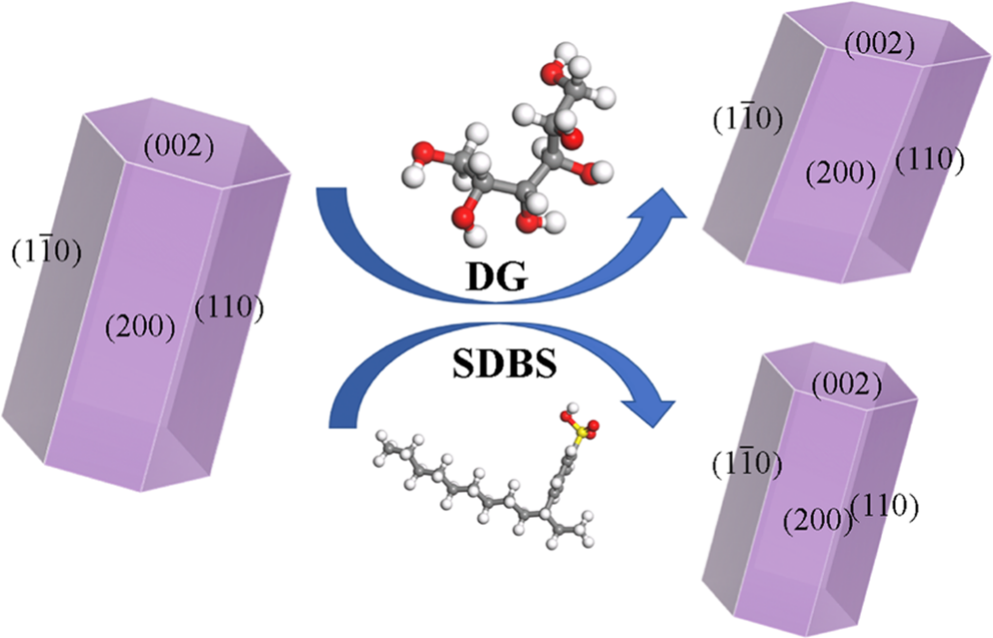
Molecular structure of SDBS and DG molecules and the modified crystal habit of CaSO_4_·0.5H_2_O by adding SDBS and DG.

Then, the theoretically modified crystal habits of CaSO_4_·0.5H_2_O with the presence of SDBS and DG are obtained based on the attachment energy model, as shown in Figure 5. The aspect ratio of the CaSO_4_·0.5H_2_O crystal decreases from 2.076 to 1.583 when DG is introduced, and SDBS has little influence on the habit of CaSO_4_·0.5H_2_O with an aspect ratio of 2.053. The DG molecule prefers to adsorb on the (002) face than the other three faces and inhibits the crystal growth along the *c* axis, leading to a smaller aspect ratio and a modified crystal habit. It is worth noting that although the interaction energy between the SDBS molecule and the (1–10) plane is stronger than that of other crystal planes, SDBS has a limited impact on the aspect ratio and morphology of phosphogypsum both experimentally and theoretically. The effect of the interaction between SDBS and the side plane (1–10) on the aspect ratio of the crystal is restricted by the other two side crystal planes (200) and (110). And the (002) plane has the greatest influence on the habit of the CaSO_4_·0.5H_2_O crystal. Interestingly, the calculated theoretical crystal habit shown in Figure 5 is consistent with the SEM results shown in Figure 4.

**Figure 6:**
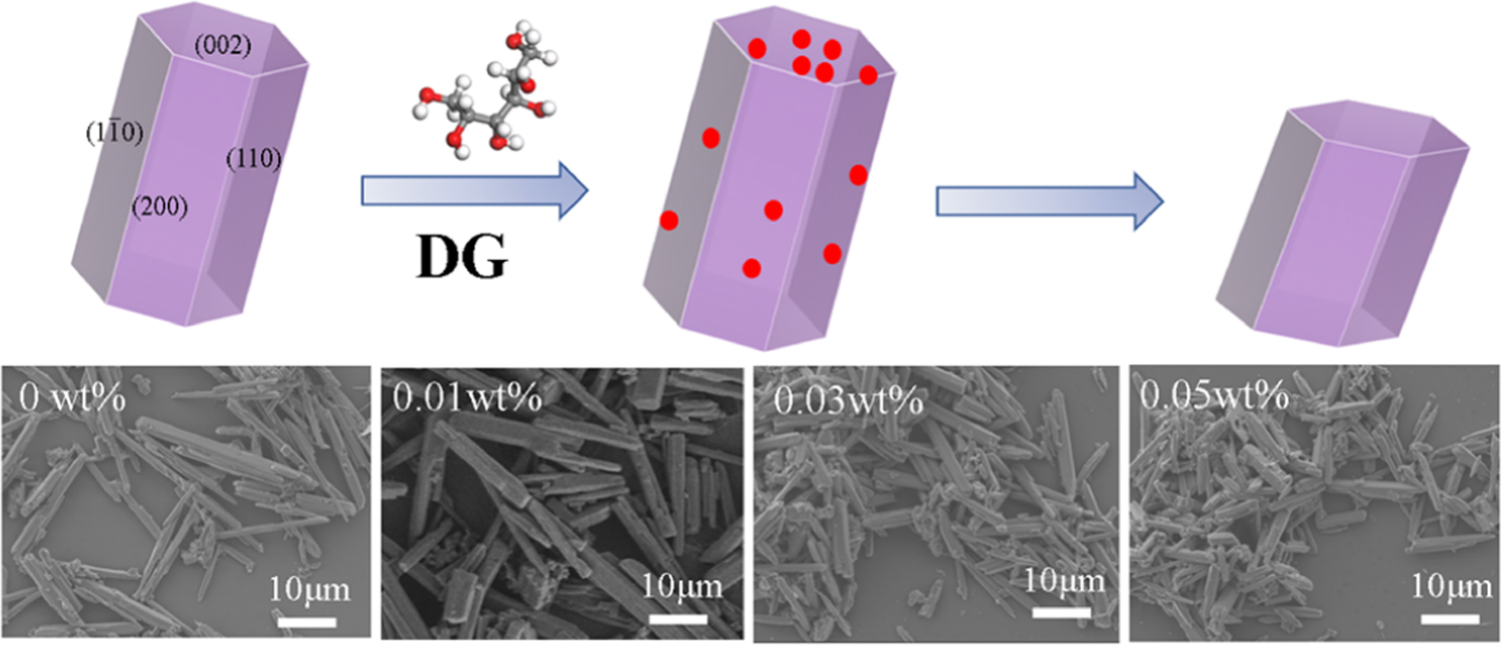
Schematic diagram of the impact of DG on the habit of CaSO_4_·0.5H_2_O and the morphologies of hemihydrate phosphogypsum with the addition of different amounts of DG.

Thus, the influence mechanism of DG on the crystallization of hemihydrate phosphogypsum is shown in Figure 6. When no additive is added, hemihydrate phosphogypsum shows a needlelike structure with a high aspect ratio. The addition of DG turns the morphology of hemihydrate phosphogypsum from needlelike to a shorter and coarser one. Based on the molecular dynamics results, the DG molecule interacts strongly with the surface of CaSO_4_·0.5H_2_O and holds the biggest interaction energy with the (002) face. The DG molecule prefers to absorb on the (002) face of CaSO_4_·0.5H_2_O especially, which strongly inhibits crystal growth along the *c* axis and thus decreases the aspect ratio. The strong interaction energy between the DG molecule and the (002) crystal face is ascribed to the hydroxyl group of the DG molecule and the exposed water molecule on the (002) face of CaSO_4_·0.5H_2_O (Figure S1). Further increasing or decreasing the dosage of DG results in little change in the morphology, as shown in Figure 6.

**Figure 7:**
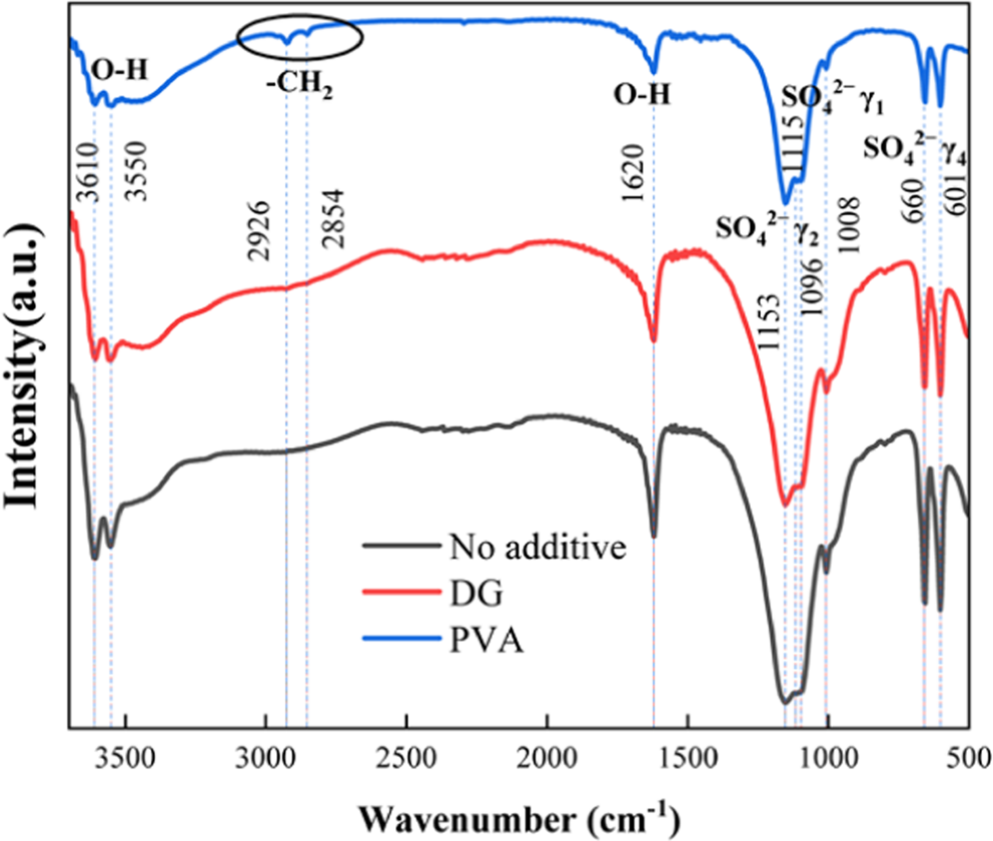
Fourier-transform infrared spectra of leaching residues with different additives.

Since there is nearly no interaction between the PVA chain and crystal planes of CaSO_4_·0.5H_2_O, how does PVA influence the crystallization behavior of hemihydrate phosphogypsum? Fourier-transform infrared and energy-dispersive spectroscopy of the leaching residue under different additives were performed to assess the role of PVA on hemihydrate phosphogypsum crystallization. The Fourier-transform infrared spectra of the obtained hemihydrate phosphogypsum with different additives are shown in Figure 7. The absorption peaks at 3610, 3550, and 1620 cm^–1^ can be associated with the vibration of O–H of crystal water and the characteristic peaks at 1008 cm^–1^ can be assigned to the characteristic frequency of γ_1_ (SO_4_^2–^) stretching; 1153, 1115, and 1096 cm^–1^ of γ_3_ (SO_4_^2–^) stretching; and 660 and 601 cm^–1^ of γ_4_ (SO_4_^2–^) stretching of the sulfate ion, respectively.^36^ The FTIR result agrees with the previous XRD results that show that CaSO_4_·0.5H_2_O is the main composition of the leaching residue. The addition of DG contributes no characteristic peaks apart from the characteristic peaks of CaSO_4_·0.5H_2_O, while the presence of PVA introduces two new characteristic peaks of the asymmetric and symmetric stretching vibrations of CH_2_ at 2926 and 2854 cm^–1^, which indicates that PVA remains in the phosphogypsum residue and may participate in the formation of a clusterlike morphology while the soluble DG enters the filtrate, as no additional characteristic infrared peaks in the leaching residue with the addition of DG were found.

**Figure 8:**
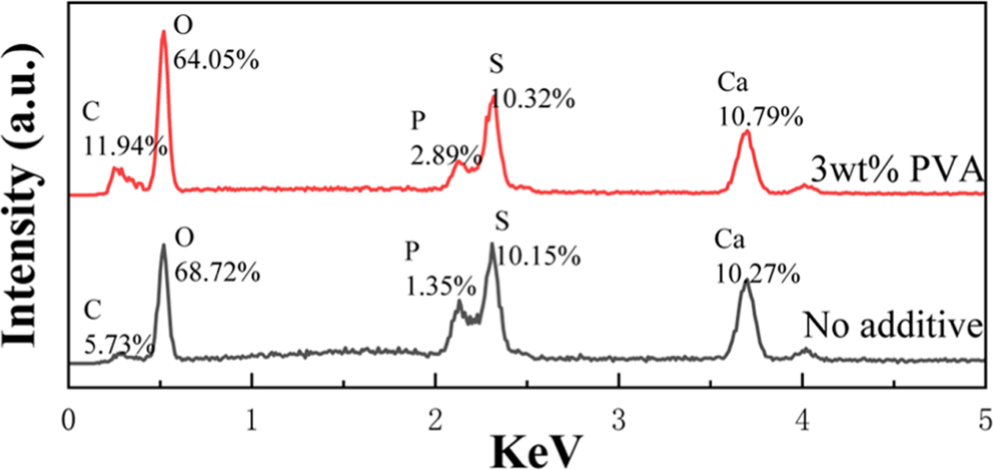
EDS patterns of hemihydrate phosphogypsum obtained without and with 3 wt % PVA.

The EDS results of hemihydrate phosphogypsum obtained with and without PVA are listed in Figure 8. The content of carbon in phosphogypsum with the addition of PVA is more than twice as much as that of hemihydrate phosphogypsum without any additives and the carbon of phosphogypsum without any additives comes from the organic matter in phosphate ore,^37^ which also indicates that PVA remains in phosphogypsum and may participate in the formation of a clusterlike morphology despite a very weak interaction with the crystal plane of CaSO_4_·0.5H_2_O (Table 2).

**Figure 9:**
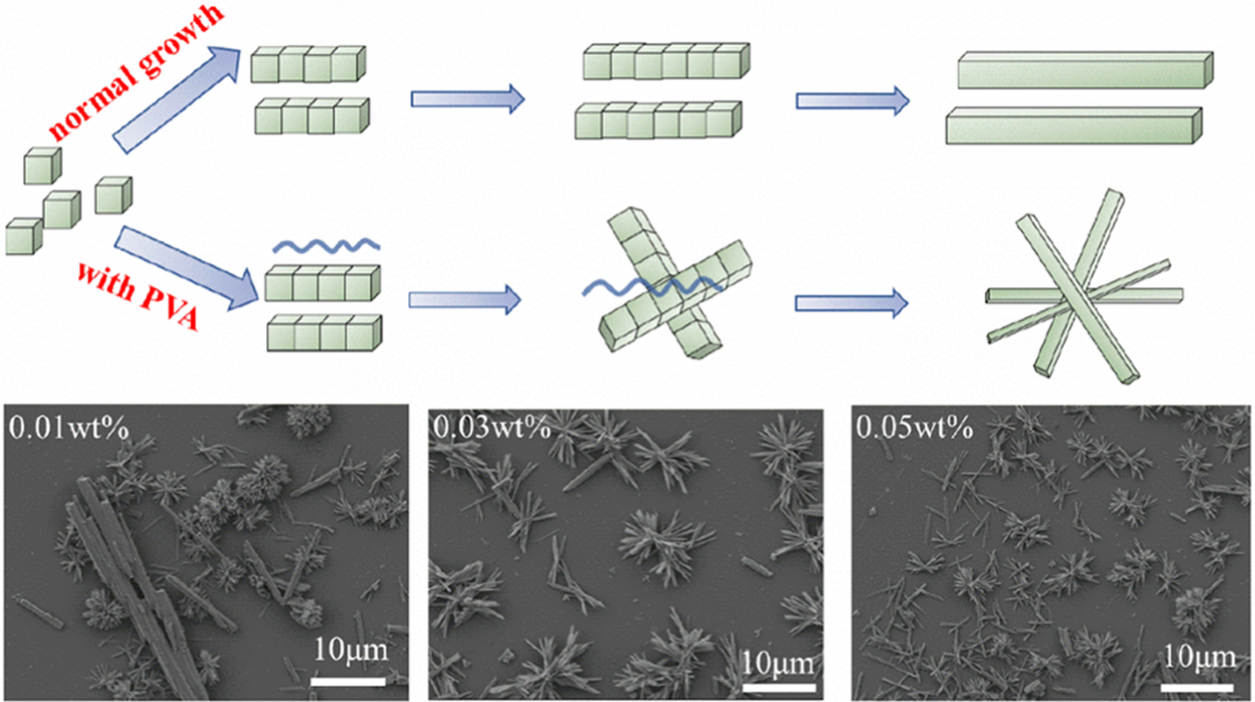
Schematic diagram of the mechanism of PVA affecting the crystallization of hemihydrate phosphogypsum and SEM images of leaching residues with the addition of different amounts of PVA.

A possible mechanism of the PVA-assisted hemihydrate phosphogypsum crystallization process is proposed in Figure 9. The PVA chain does not absorb on any crystal face of the CaSO_4_·0.5H_2_O crystal, which is confirmed by the molecular dynamics results (Table 2). However, it actually modifies the crystallization behavior of CaSO_4_·0.5H_2_O by aggregating dispersed needlelike crystal grains into clusters (Figure 4f). Based on the FTIR and EDS results, the acid-insoluble PVA remains in phosphogypsum, and PVA may act as a bridge so that the CaSO_4_·0.5H_2_O grains grow together into a cluster structure before growing larger, as the individual grains are finer with the presence of PVA. When only 0.01 wt % PVA is added to the leaching system, the crystal aggregation of hemihydrate phosphogypsum is insufficient, and single needlelike phosphogypsum still exists, as shown in Figure 9. Nearly all of the phosphogypsum grains aggregate into a clusterlike morphology after increasing the concentration of PVA to 0.03 and 0.05 wt %, which strongly convinces the role of PVA in morphology modification of hemihydrate phosphogypsum.

### 3.4 Relationship between Phosphogypsum Morphology and Filtration Strength

The filter cake of the leaching slurries’ filtration process is formed through the entrapment of phosphogypsum crystals by the filter cloth and is composed of numerous overlapped and staggered small phosphogypsum crystals. The pores formed by crystal stacking are the channels of the filtrate in the filtration process, and the filter cake porosity becomes an important criterion in determining the filtration strength. Obviously, the more and larger the pores in the filter cake, the more conducive it is to the smooth passage of the phosphoric acid filtrate and the faster is the filtration strength achieved.

**Figure 10:**
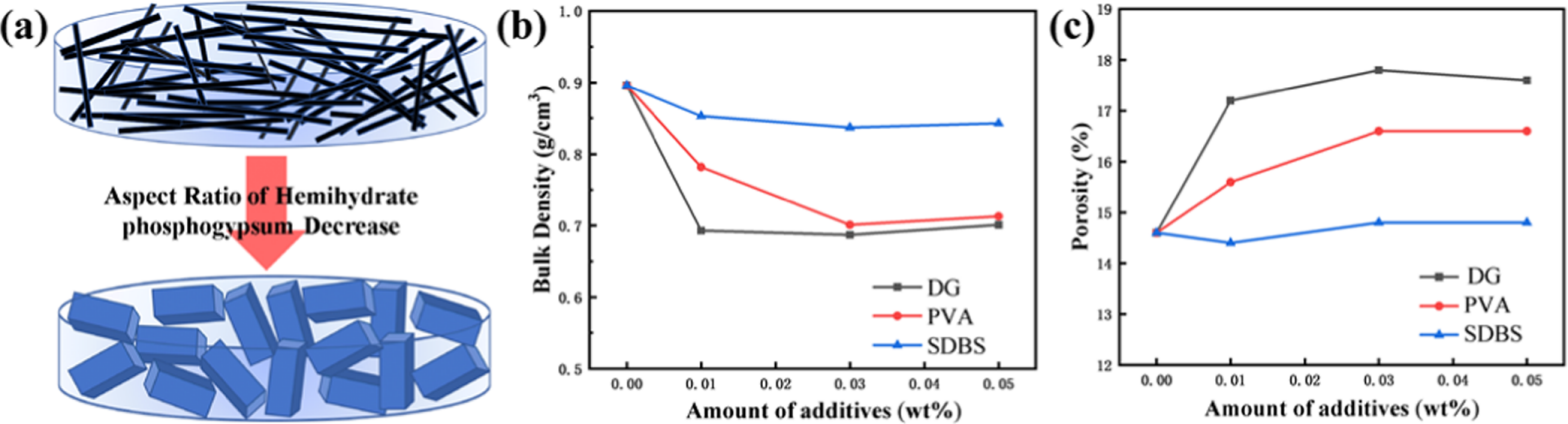
(a) Stacking diagram of phosphogypsum grains with different aspect ratios in the filter cake and the (b) porosity and (c) bulk density of the leaching residue with the addition of different additives.

The pores in the filter cake are formed due to the spatial repulsion between phosphogypsum crystals, as shown in Figure 10a, and the pore size of the phosphogypsum filter cake varies with different crystal habits, which is confirmed by the bulk density of hemihydrate phosphogypsum shown in Figure 10b and the porosity of the filter cake shown in Figure 10c. The modified morphologies by DG and PVA are beneficial to increase the volume of stacking pores, which is confirmed by the porosity results. It is shown that the lower bulk density of phosphogypsum and larger porosity of the filter cake are achieved when DG and PVA are added, which is beneficial to a smoother filter path and improves the filtration strength, as shown in Figure 3.

## 4 Conclusions

To sum up, in order to improve the filtration strength of hemihydrate phosphogypsum produced in the first stage of HH–DH WPA, the effects of additives on the particle size and the crystal morphology of hemihydrate phosphogypsum are systematically studied. The addition of DG and PVA has a great impact on the crystal habit of hemihydrate phosphogypsum. The molecular dynamics results show that DG reduces the aspect ratio of phosphogypsum through the strong interaction with the (002) crystal face of CaSO_4_·0.5H_2_O so that the needle-shaped phosphogypsum eventually becomes shorter and coarser. The addition of PVA changed the growth behavior of hemihydrate phosphogypsum, which makes small phosphogypsum crystals aggregate to form a clusterlike structure. The phosphogypsum morphology modified by DG and PVA makes a smaller bulk density and a larger porosity of the filter cake. Our work shows that the modified morphologies by DG and PVA enhance the filtration strength of phosphogypsum, and the enhanced filtration strength would reduce the water content of hemihydrate phosphogypsum and relieve the storage pressure of the phosphogypsum slag dump, which is meaningful to the clean production and process emission reduction of the phosphorus chemical industry.

## Supporting Information Available

Atomic structures of the (002), (200), (110), and (1–10) crystal faces of CaSO_4_·0.5H_2_O; optical images and XRD patterns of the leaching residue of the first 10 min of HH–DH WPA (PDF)

## References

[ref1] Mu X.; Zhu G.; Li X.; Li S.; Gong X.; Li H.; Sun G. Effects of Impurities on CaSO4 Crystallization in the Ca(H2PO4)2-H2SO4-H3PO4-H2O System. ACS Omega 2019, 4 (7), 12702–12710. 10.1021/acsomega.9b01114. 31460392 PMC6682056

[ref2] Ma C.; Wu Y. X.; Jin F.; Li X.; Hu B. Current Status and Prospect of Industrial Phosphoric Acid Production. Chem. Eng. 2013, 41 (6), 74–78. 10.3969/j.issn.1005-9954.2013.06.018.

[ref3] Peng Y.; Zhu Z.; Braatz R. D.; Myerson A. S. Gypsum Crystallization during Phosphoric Acid Production: Modeling and Experiments Using the Mixed-Solvent-Electrolyte Thermodynamic Model. Ind. Eng. Chem. Res. 2015, 54 (32), 7914–7924. 10.1021/acs.iecr.5b01763.

[ref4] Gobbitt J. M. Yara Hemihydrate (HH) and Hemidihydrate (HDH) Processes for Phosphoric Acid Production. Procedia Eng. 2012, 46, 143–153. 10.1016/j.proeng.2012.09.457.

[ref5] Wang B.; Yang L.; Luo T.; Cao J. Study on the Kinetics of Hydration Transformation from Hemihydrate Phosphogypsum to Dihydrate Phosphogypsum in Simulated Wet Process Phosphoric Acid. ACS Omega 2021, 6 (11), 7342–7350. 10.1021/acsomega.0c05432. 33778247 PMC7992066

[ref6] Van Der Sluis S.; Witkamp G. J.; Van Rosmalen G. M. Crystallization of Calcium Sulfate in Concentrated Phosphoric Acid. J. Cryst. Growth 1986, 79 (1–3), 620–629. 10.1016/0022-0248(86)90529-4.

[ref7] Guan B.; Shen Z.; Wu Z.; Yang L.; Ma X. Effect of PH on the Preparation of α-Calcium Sulfate Hemihydrate from FGD Gypsum with the Hydrothermal Method. J. Am. Ceram. Soc. 2008, 91 (12), 3835–3840. 10.1111/j.1551-2916.2008.02729.x.

[ref8] Li Z.; Liu Y.; Xing D.; Hua Q.; Wang B.; Liu L.; Tang J. Effect of Maleic Acid and PH on the Preparation of α-Calcium Sulfate Hemihydrate from Phosphogypsum in Mg(NO3)2 Solution. J. Mater. Cycles Waste Manage. 2022, 24 (1), 143–154. 10.1007/s10163-021-01304-6.

[ref9] Li F.; Liu J.; Yang G.; Pan Z.; Ni X.; Xu H.; Huang Q. Effect of PH and Succinic Acid on the Morphology of α-Calcium Sulfate Hemihydrate Synthesized by a Salt Solution Method. J. Cryst. Growth 2013, 374, 31–36. 10.1016/j.jcrysgro.2013.03.042.

[ref10] Abdelouahhab M.; Manar S.; Benhida R. Optimization and Evaluation of the Effect of Impurities on Phosphoric Acid Process Performance Using Design of Experiments. Results Eng. 2022, 15, 100501 10.1016/j.rineng.2022.100501.

[ref11] Titiz-Sargut S.; Sayan P.; Avci B. Influence of Citric Acid on Calcium Sulfate Dihydrate Crystallization in Aqueous Media. Cryst. Res. Technol. 2007, 42 (2), 119–126. 10.1002/crat.200610783.

[ref12] Kruger A.; Focke W. W.; Kwela Z.; Fowles R. Materials and Interfaces: Effect of Ionic Impurities on the Crystallization of Gypsum in Wet-Process Phosphoric Acid. Ind. Eng. Chem. Res. 2001, 40 (5), 1364–1369. 10.1021/ie000478b.

[ref13] El-Shall H.; Abdel-Aal E. A.; Moudgil B. M. Effect of Surfactants on Phosphogypsum Crystallization and Filtration during Wet-Process Phosphoric Acid Production. Sep. Sci. Technol. 2000, 35 (3), 395–410. 10.1081/SS-100100164.

[ref14] Prisciandaro M.; Olivieri E.; Lancia A.; Musmarra D. Gypsum Precipitation from an Aqueous Solution in the Presence of Nitrilotrimethylenephosphonic Acid. Ind. Eng. Chem. Res. 2006, 45 (6), 2070–2076. 10.1021/ie050615a.

[ref15] Polat S.; Sayan P. Determination of the Effects of Carboxylic Acids on Calcium Sulfate Dihydrate Crystallization. Chem. Eng. Technol. 2017, 40 (7), 1354–1361. 10.1002/ceat.201600525.

[ref16] Abdel-Aal E. A.; Mahmoud M. M. H.; El-Shall H.; Ismail A. K. Increasing the Filtration Rate of Phospho-Gypsum Using Surfactant. Hydrometallurgy 2007, 85 (1), 53–58. 10.1016/j.hydromet.2006.08.004.

[ref17] Khan M. A. S.; Vijayalakshmi R.; Singh A.; Nandi A. K.; Talawar M. B. Morphology of Ammonium Perchlorate in the Presence of Ethylene Glycol as an Additive: A First Principle Study. CrystEngComm 2019, 21 (48), 7519–7527. 10.1039/C9CE01262A.

[ref18] Han D.; Wang Y.; Yang Y.; Gong T.; Chen Y.; Gong J. Revealing the Role of a Surfactant in the Nucleation and Crystal Growth of Thiamine Nitrate: Experiments and Simulation Studies. CrystEngComm 2019, 21 (23), 3576–3585. 10.1039/C9CE00325H.

[ref19] Singh M. K.; Banerjee A. Role of Tailor-Made Additives in Controlling Vapour Growth Asymmetry along the Polar Axis of α-Resorcinol Crystals: A Molecular-Scale Study. CrystEngComm 2018, 20 (26), 3673–3687. 10.1039/C8CE00118A.

[ref20] Rashad M. M.; Mahmoud M. H. H.; Ibrahim I. A.; Abdel-Aal E. A. Crystallization of Calcium Sulfate Dihydrate under Simulated Conditions of Phosphoric Acid Production in the Presence of Aluminum and Magnesium Ions. J. Cryst. Growth 2004, 267 (1–2), 372–379. 10.1016/j.jcrysgro.2004.03.060.

[ref21] Mahmoud M. H. H.; Rashad M. M.; Ibrahim I. A.; Abdel-Aal E. A. Crystal Modification of Calcium Sulfate Dihydrate in the Presence of Some Surface-Active Agents. J. Colloid Interface Sci. 2004, 270 (1), 99–105. 10.1016/j.jcis.2003.09.023. 14693140

[ref22] Hou S.; Wang J.; Wang X.; Chen H.; Xiang L. Effect of Mg2+ on Hydrothermal Formation of α-CaSO 4·0.5H2O Whiskers with High Aspect Ratios. Langmuir 2014, 30 (32), 9804–9810. 10.1021/la502451f. 25089651

[ref23] Fan H.; Song X.; Liu T.; Xu Y.; Yu J. Effect of Al3+ on Crystal Morphology and Size of Calcium Sulfate Hemihydrate: Experimental and Molecular Dynamics Simulation Study. J. Cryst. Growth 2018, 495, 29–36. 10.1016/j.jcrysgro.2018.05.013.

[ref24] Li X.; Xu D.; Yang X.; Wu J.; Wang B.; Li X.; Wang X.; Zhang Z. Influence of Aluminum on Morphologies and Crystallization Kinetics of Hemihydrate Calcium Sulfate in the Hemihydrate Process of Phosphoric Acid Production. Ind. Eng. Chem. Res. 2022, 61 (28), 10069–10077. 10.1021/acs.iecr.2c01827.

[ref25] Mao X.; Song X.; Lu G.; Sun Y.; Xu Y.; Yu J. Control of Crystal Morphology and Size of Calcium Sulfate Whiskers in Aqueous HCl Solutions by Additives: Experimental and Molecular Dynamics Simulation Studies. Ind. Eng. Chem. Res. 2015, 54 (17), 4781–4787. 10.1021/acs.iecr.5b00585.

[ref26] Yang L.; Cao J.; Luo T. Effect of Mg2+, Al3+, and Fe3+ Ions on Crystallization of Type α Hemi-Hydrated Calcium Sulfate under Simulated Conditions of Hemi-Hydrate Process of Phosphoric Acid. J. Cryst. Growth 2018, 486, 30–37. 10.1016/j.jcrysgro.2018.01.014.

[ref27] Liu R.; Long Y.; Zhou Y.; Liu Z.; Liu X.; Huo X.; Xie Z.; Tao C. Rigid-Flexible Combined Impeller Enhancement in Leaching of Phosphate Rock: A Kinetics Study. ACS Omega 2021, 6 (48), 33206–33214. 10.1021/acsomega.1c05836. 34901672 PMC8656210

[ref28] Beck R.; Häkkinen A.; Malthe-Sørenssen D.; Andreassen J. P. The Effect of Crystallization Conditions, Crystal Morphology and Size on Pressure Filtration of l-Glutamic Acid and an Aromatic Amine. Sep. Purif. Technol. 2009, 66 (3), 549–558. 10.1016/j.seppur.2009.01.018.

[ref29] Bezou C.; Nonat A.; Mutin J. C.; Nørlund Christensen A.; Lehmann M. S. Investigation of the Crystal Structure of γ-CaSO4, CaSO4 · 0.5 H2O, and CaSO4 · 0.6 H2O by Powder Diffraction Methods. J. Solid State Chem. 1995, 117, 165–176. 10.1006/jssc.1995.1260.

[ref30] Hartman P.; Bennema P. The attachment energy as a habit controlling factor: I. Theoretical Considerations. J. Cryst. Growth 1980, 49, 145–156. 10.1016/0022-0248(80)90075-5.

[ref31] Hartman P. The attachment energy as a habit controlling factor: II. Application to Anthracene, Tin Tetraiodide and Orthorhombic Sulphur. J. Cryst. Growth 1980, 49, 157–165. 10.1016/0022-0248(80)90076-7.

[ref32] Hartman P. The Attachment Energy as a Habit Controlling Factor. III. Application to Corundum. J. Cryst. Growth 1980, 49 (1), 166–170. 10.1016/0022-0248(80)90077-9.

[ref33] Lu J. J.; Ulrich J. An Improved Prediction Model of Morphological Modifications of Organic Crystals Induced by Additives. Cryst. Res. Technol. 2003, 38 (1), 63–73. 10.1002/crat.200310008.

[ref34] McDonald M. A.; Salami H.; Harris P. R.; Lagerman C. E.; Yang X.; Bommarius A. S.; Grover M. A.; Rousseau R. W. Reactive Crystallization: A Review. React. Chem. Eng. 2021, 6 (3), 364–400. 10.1039/D0RE00272K.

[ref35] Tang Y.; Gao J. Investigation of the Effects of Sodium Dicarboxylates on the Crystal Habit of Calcium Sulfate α-Hemihydrate. Langmuir 2017, 33 (38), 9637–9644. 10.1021/acs.langmuir.7b02380. 28859476

[ref36] Chen Q.; Jia C.; Li Y.; Xu J.; Guan B.; Yates M. Z. α-Calcium Sulfate Hemihydrate Nanorods Synthesis: A Method for Nanoparticle Preparation by Mesocrystallization. Langmuir 2017, 33 (9), 2362–2369. 10.1021/acs.langmuir.7b00013. 28161955

[ref37] Zhou Y.; Zheng G.; Long Y.; Liu Z.; Tao C.; Liu R. Advanced Oxidation Processes for Wet-Process Phosphoric Acid: Enhanced Phosphorus Recovery and Removal of Organic Matters. Hydrometallurgy 2022, 210, 105842 10.1016/j.hydromet.2022.105842.

