# Supplementary material for: Role of Additives: Modified Hemihydrate Phosphogypsum Morphology and Enhanced Filtration Performance of Wet-Process Phosphoric Acid
Source: ACS Omega. 2023 Dec 1;8(49):47295–303. doi: 10.1021/acsomega.3c08259 (PMC10720274; doi:10.1021/acsomega.3c08259)
Supplement: Supplementary file 1 — ao3c08259_si_001.pdf [file ao3c08259_si_001.pdf]

Supporting Information for

The role of additives: modified hemihydrate phosphogypsum morphology and enhanced filtration performance of wet-process phosphoric acid

Xuejian Huo[1,2], Lanfeng Guo[1,2], Renlong Liu[1,2]*, Changyuan Tao[1,2], Benjun Xi[3]

1. College of Chemistry and Chemical Engineering, Chongqing University, Chongqing, 400044, China
2. State Key Laboratory of Coal Mine Disaster Dynamics and Control, Chongqing University, Chongqing, 400044, China
3. Hubei Three Gorges Laboratory, Yichang, 443007, China

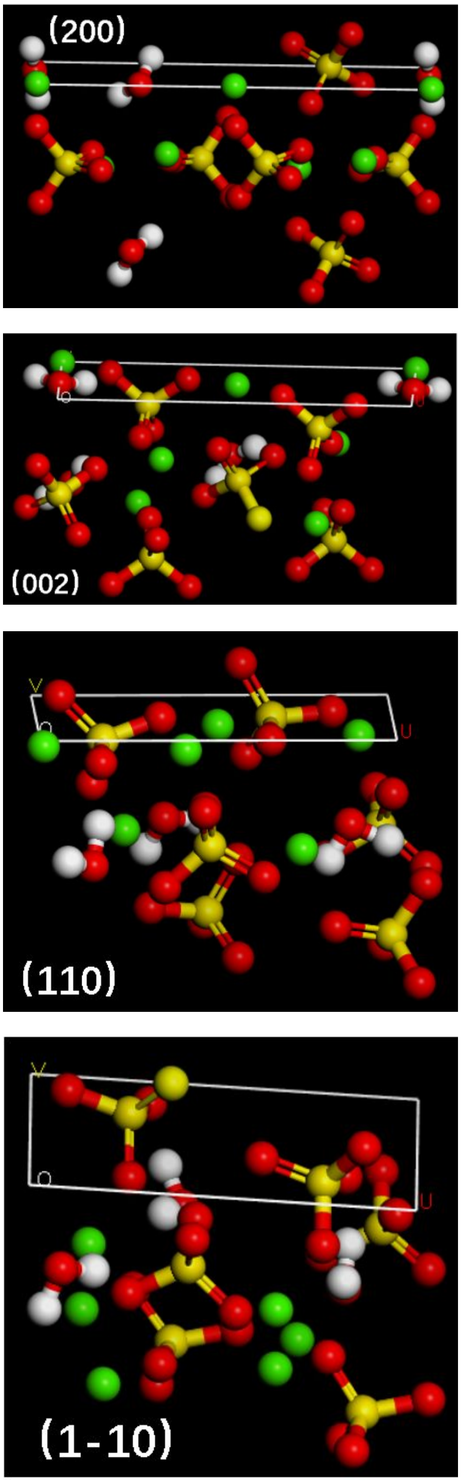

Fig.S1 The atomic structure of the (002), (200), (110), (1-10) crystal face of $CaSO_4 \cdot 0.5H_2O$.

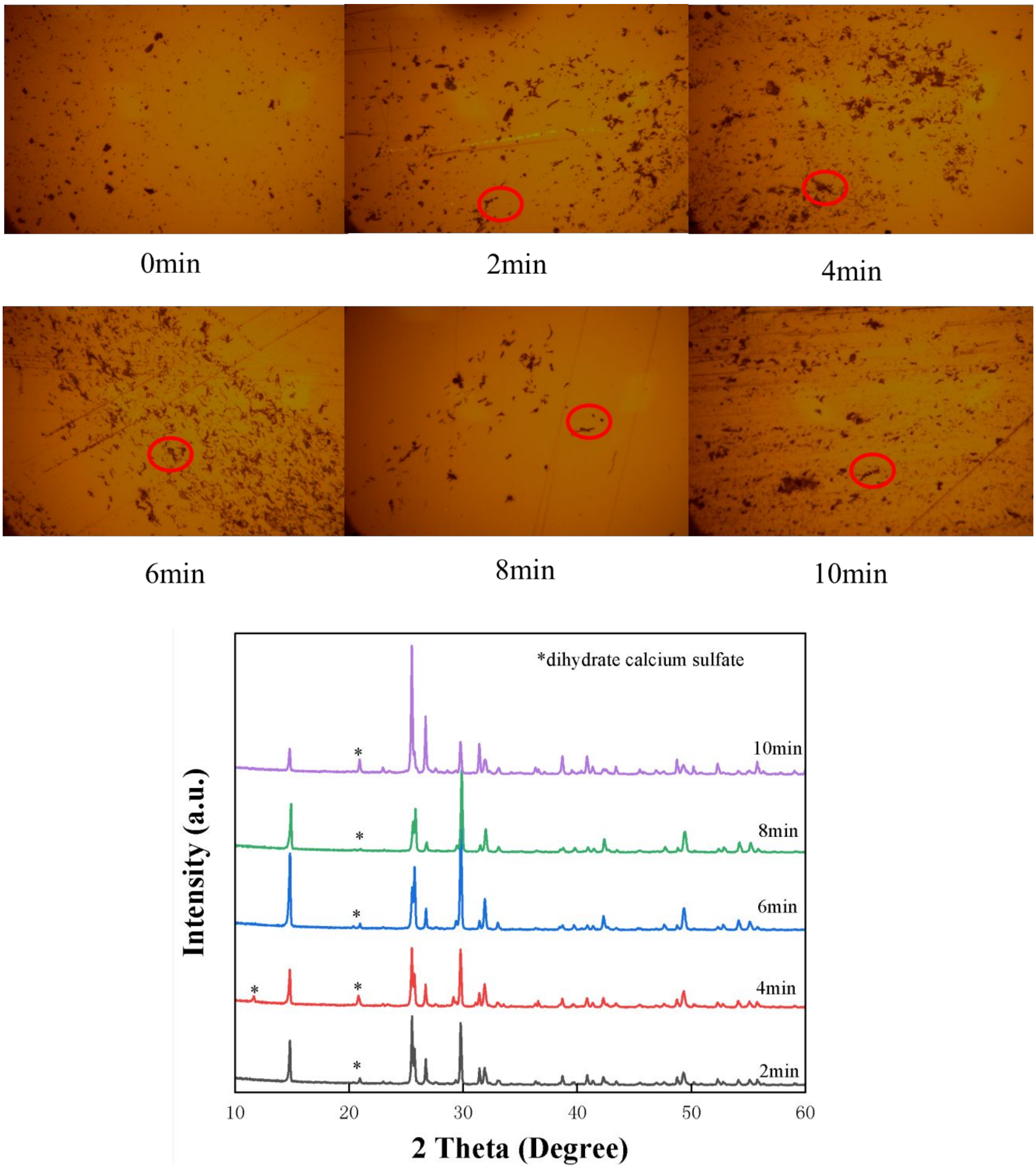

Fig.S2 The optical images and XRD patterns of leaching residue of the first ten minutes HH-DH WPA.

.
